# The Genetic Diversity of Horse Native Breeds in Russia

**DOI:** 10.3390/genes14122148

**Published:** 2023-11-28

**Authors:** Mikhail Atroshchenko, Natalia Dementieva, Yuri Shcherbakov, Olga Nikolaeva, Anastasiia Azovtseva, Anna Ryabova, Elena Nikitkina, Oksana Makhmutova, Andrey Datsyshin, Viktor Zakharov, Alexander Zaitsev

**Affiliations:** 1 All-Russian Research Institute of Horse Breeding (ARRIH), Ryazan Region, Rybnovskij District, Divovo 391105, Russia; atromiks-77@mail.ru (M.A.); 69-mahaon@mail.ru (O.M.); dacyshin@yandex.ru (A.D.); e-slotina@mail.ru (V.Z.); amzaitceff@mail.ru (A.Z.); 2 Russian Research Institute of Farm Animal Genetics and Breeding—Branch of the L.K. Ernst Federal Research Center for Animal Husbandry, 55A, Moskovskoye Sh., Tyarlevo, St. Petersburg, Pushkin 196625, Russia; yura.10.08.94.94@mail.ru (Y.S.); trantoburito@mail.ru (O.N.); ase4ica15@mail.ru (A.A.); aniuta.riabova2016@yandex.ru (A.R.); nikitkinae@yandex.ru (E.N.)

**Keywords:** equus caballus, breeds, Single Nucleotide Polymorphisms (SNPs), genetic diversity, runs of homozygosity

## Abstract

Horses were domesticated later than other farm animals. Horse breeds have been selectively developed by humans to satisfy different needs and purposes. The factory and indigenous breeds are of particular interest, having been bred in purity for many centuries without the addition of foreign blood. Data from 31 stud farms, as well as ranches, located in fifteen regions of the Russian Federation were used in this work. DNA was sampled from 102 stallions of 11 breeds: Arabian, Akhal-Teke, Don, Orlov Trotter, Vladimir Heavy Draft, Russian Heavy Draft, Soviet Heavy Draft, Kabardin, Yakut, Tuva, and Vyatka. Data on the origin of each animal from which the material was collected were taken into account. DNA genotyping was carried out using GGP Equine 70 k ^®^ array chips (Thermo Fisher Scientific, USA). Genetic diversity of horse breeds was estimated using Admixture 1.3. and PLINK 1.9 software. FROH inbreeding was computed via the R detectRUNS package. The minimum length for ROH was set at 1 Mb to reduce the occurrence of false positives. We conducted PCA analysis using PLINK 1.9, and used the ggplot2 library in R for visualizing the results. Indigenous equine breeds, such as Vyatka, Tuva, and Yakut, are very hardy, and well adapted to local environmental and climatic conditions. They are employed as draft power, as well as for milk and meat. Both the Akhal-Teke breed and the Arabian breed have retained a minimum effective population size over many generations. We note significant accumulations of homozygosity in these breeds. In equestrian sports, performance is a top priority. ADMIXTURE and PCA analyses showed similarities between Don equine breeds and Kabardin, as well as some Arabian breed animals. Earlier research indicated the presence of thoroughbred traits in Don stallions. The Orlov Trotter breed stands out as a separate cluster in the structural and PCA analyses. Considering the small population size of this breed, our study found high FROH in all tested animals. The general reduction in the diversity of the horse breed gene pool, due to numerous crosses for breed improvement with thoroughbreds, has lead to a decline in the differences between the top sporting breeds. Our study presents new opportunities for exploring the genetic factors that influence the formation of adaptive traits in indigenous breeds, and for finding ways to preserve genetic diversity for effective population reproduction.

## 1. Introduction

Horses are one of the most important domesticated animals that have played a huge role in the history of mankind. Despite the fact that their domestication commenced much later than that of other domestic animals, the diversity of their breeds is remarkable [1]. These variations are due to the different purposes for which horses have been intensively used in human life. As a result of strict selective breeding for certain traits, several types of equines have been formed. Conventionally, they are divided into three categories. The first includes horses primarily used for riding, the second includes horses used for draught [2], and the third includes horses used for milk or meat. Certain indigenous breeds were utilised for both dairy and meat production [3,4]. The development of horse breeds was particularly influenced by the widespread settlement of people around the globe, and the ability of horses to adapt to different climates and conditions of use [5]. Horses have been used in manufacturing, as a means of transport and communication between nations, in wars, and even as a symbol of wealth. Stallions possessing distinctive traits were highly valued and selectively bred within local communities through inbreeding among closely related individuals, leading to a substantial decrease in genetic variation among the breeds now recognized as thoroughbreds [6]. These breeding methods have resulted in a loss of adaptive plasticity to husbandry conditions, making it challenging to implement breeds on a broad scale. There were indigenous breeds everywhere that were not efficient enough in a rapidly developing society that required a combination of high working qualities and external phenotypic characteristics. As the result of crossing, breeds with thoroughbred stallions in their pedigree have emerged. In contemporary times, enhancing tribal resources using such breeds has resulted in a considerable loss of breed differentiation. Reproduction of some horse breeds has almost eliminated genetic diversity, resulting in the loss of the unique quality of these breeds. Technological advancements have superseded horses, which had occupied a niche for centuries that had been exploited by humans. However, the Russian Federation, with its vast territory, diverse natural conditions, and rich equine breeding history, owns numerous horse breeds of indigenous origin [7]. Recently, there have been efforts to evaluate the genetic variation of breeds bred within Russia’s borders [8], but these were Stud breeds that showed a reduction in the uniqueness of some breeds, though there is still no clear picture of all the genetic variability in native horse breeds.

This study presents both Russian indigenous and production breeds, which have been inbreeding for centuries, avoiding the infusion of blood from other breeds. The evaluation of genetic diversity using a medium-density chip for indigenous and certain commercial equine varieties is important for the preservation of the world’s equine genetic resources.

## 2. Materials and Methods

### 2.1. Ethics Statement

The principles of laboratory animal care were followed, and all procedures were conducted according to the ethical guidelines of the All-Russian Research Institute of Horse Breeding. The protocol was approved by the Commission for the Control of the Keeping and Use of Experimental Animals (Commission on Bioethics) of the All-Russian Research Institute of Horse Breeding (Protocol Number: 2022/11/3), and the Law of the Russia Federation on Veterinary Medicine No. 4979-1 (14 May 1993).

### 2.2. DNA Extraction

Materials for this study were collected from 31 equine breeding farms situated within 15 regions of the Russian Federation. SNP variations were analysed in 102 specimens from 11 indigenous and stud equine breeds.

**Figure 1:**
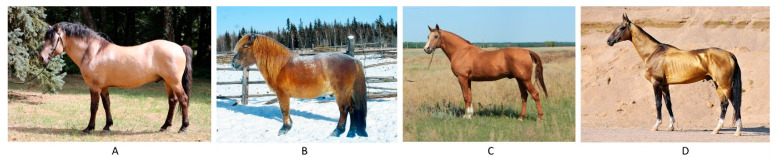
Examples of individual stallions used in the present study: (**A**) List, Vyatka breed; (**B**) Megezh, Yakut breed; (**C**) Datchanin, Don breed; (**D**) Dagat, Akhal-Teke breed.

DNA isolation was carried out from hair follicles in FSBSI All-Russian Research Institute of Horse Breeding of the Ryazan region related to Core Shared Research Facilities (CSRF) and Large-Scale Research Facilities (LSRF). The laboratory work was performed using the Biolabmix kit (Russia). DNA was isolated from 102 stallions of 11 breeds: Arabian (AR, n = 11), Akhal-Teke (AH, n = 12), Don (D, n = 11), Orlov Trotter (OR, n = 13), Vladimir Heavy Draft (VH, n = 2), Russian Heavy Draft (RH, n = 4), Soviet Heavy Draft (SH, n = 3), Kabardin (K, n = 14), Yakut (YA, n = 16), Tuva (T, n = 2), Vyatka (V, n = 14) breeds. The provenance of the animals from which the material was derived was also taken into account. Examples of the animals used in the study are shown in Figure 1. Known aboriginal Russian breeds included Yakut (YA), Tuva (T), and Vyatka (V) breeds.

### 2.3. Genotyping DNA Samples

DNA genotyping was undertaken using GGP Equine 70 k ^®^ array chips (Thermo Fisher Scientific, Waltham, MA, USA). DNA samples with genotyping quality exceeding 98% at SNP loci were chosen, and SNP selection was implemented using the PLINK 1.9 programme [9].

### 2.4. Statistical Analysis and Visualization

The genomic architecture of horse breeds was calculated using Admixture 1.3 software [10], and graphically visualised in the pophelper package of the R package of the R Studio 1.3.1093 software [11]. Cross-validation (CV) errors determined the most probable number of ancestral clusters (K) for K values ranging from 2 to 12.

Expected (He) and observed (Ho) heterozygosity values calculated from the PLINK 1.9 programme [12] produced estimations of genetic diversity.

FROH-based consanguinity was computed through the RDetectRUNS software. The length threshold for observed homozygosity regions (ROHs) was set to 1 Mb to mitigate the occurrence of false positives [13].

## 3. Results

### 3.1. Genetic Diversity and Genomic Variability in Horse Breeds

**Figure 2:**
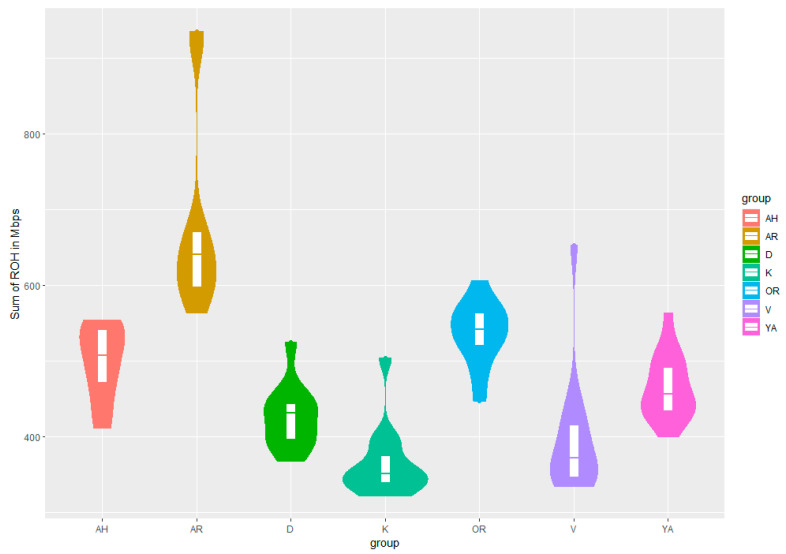
Plot of mean, quartiles, and frequency (plot width) based on the ROH of the coefficient of inbreeding (FROH) for each breed group. Breed designations: Arabian (AR), Akhal-Teke (AH), Don (D), Orlov Trotter (OR), Kabardin (K), Yakut (YA), Vyatka (V) breeds.

Based on the results of SNP genotyping, values of FIS, Ho, He, and ROH metrics were generated as the main parameters characterizing the state of genomic diversity of studied breeds (Table 1, Figure 2).

As a low number of individuals represented some horse breeds in the complete sample, we selected seven populations for additional calculations. From these populations, at minimum, ten samples were gathered and analysed. Heterozygosity values for the chosen equine breeds are presented in Table 1. The Don (0.325, 0.342) and Kabardin (0.328, 0.342) breeds showed the highest expected heterozygosity and heterozygosity observed, whereas the Yakut breed revealed the lowest (0.288, 0.298).

The Arabian and Yakut horses had the highest FIS (−0.027), and the Vyatka horses had the lowest (−0.054). The obtained data (Table 1) indicated a significant level of inbreeding in Arabian and Yakut breeds. Figure 2 shows plot of mean, quartiles, and frequency (plot width) based on the ROH of the coefficient of inbreeding (FROH) for each breed group. Maximum FROH was observed in Arabian horses. Nevertheless, the high FROH was found in individuals of Vyatka breed. High FROH is associated with the utilisation of a limited number of stallions. Minimal FROH was noted from Kabardin horses, where thoroughbred stallions are used for breed improvement.

### 3.2. Effective Population Size

**Figure 3:**
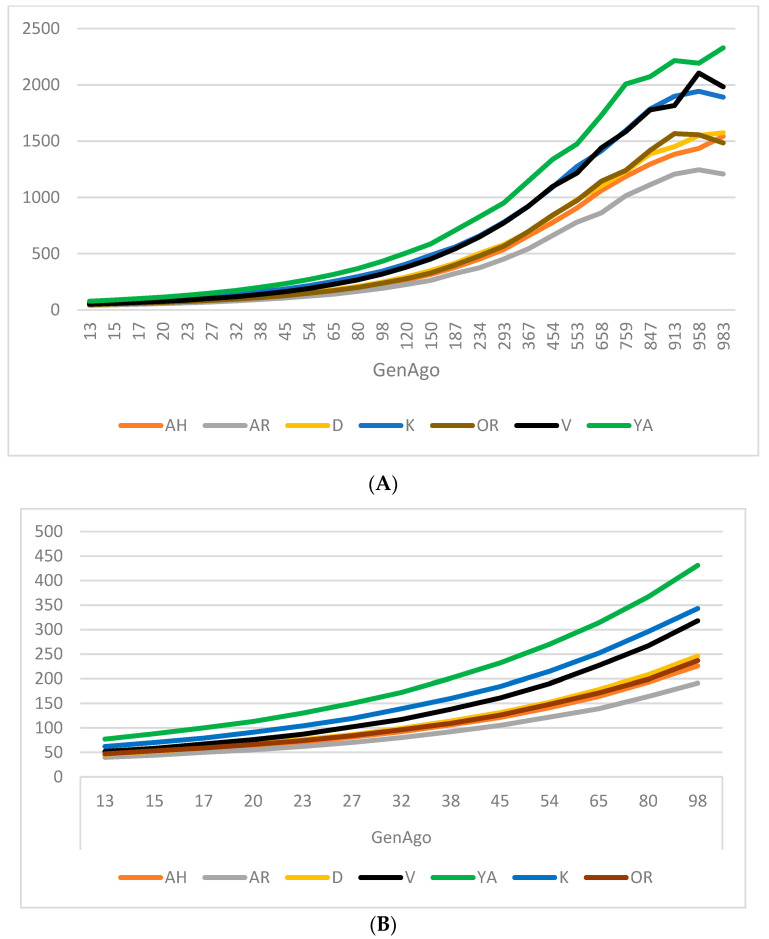
Effective population (Ne) size in 7 horse breeds: (**A**) Ne plotted against generations in the past at 983 generations; (**B**) Ne plotted against generations in the past, truncated at 98 generations.

For estimating the breed’s development dynamics, we calculated the effective population size through the downward generations (Figure 3A,B). The largest effective population size in the Yakut breed is evident in both recent and ancestral history. The minimum number of individuals can be observed within the Arabian, Akhal-Teke, Orlov Trotter, and Don breeds. This might be explained through the limited usage of stud-stallions Kabardin and Vyatka horses occupying an intermediate position in relation to the studied breeds. Crossbreeding with improved breeds has affected the ancestral populations of these breeds, resulting in improved sporting qualities in the Kabardin breed, and has been performed to restore the gene pool.

### 3.3. ADMIXTURE and PCA Analysis

**Figure 4:**
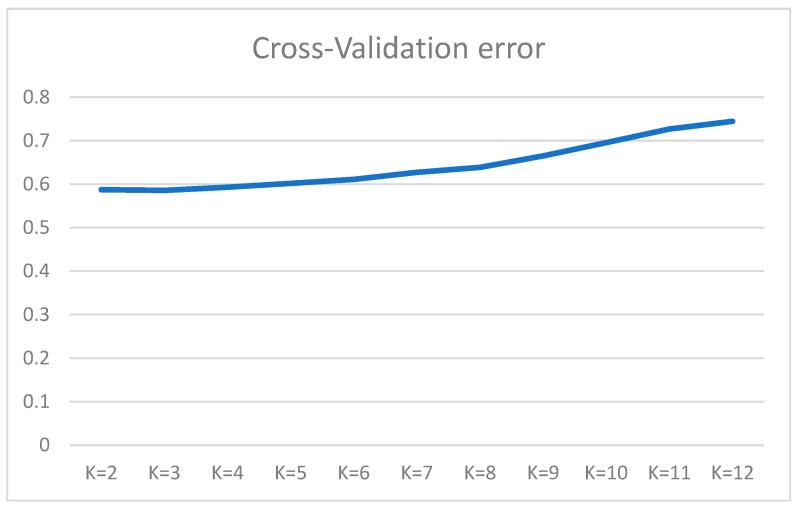
A graph obtained through calculating the number of ancestral clusters (K) from 2 to 12 based on cross-validation errors (CV% error).

**Figure 5:**
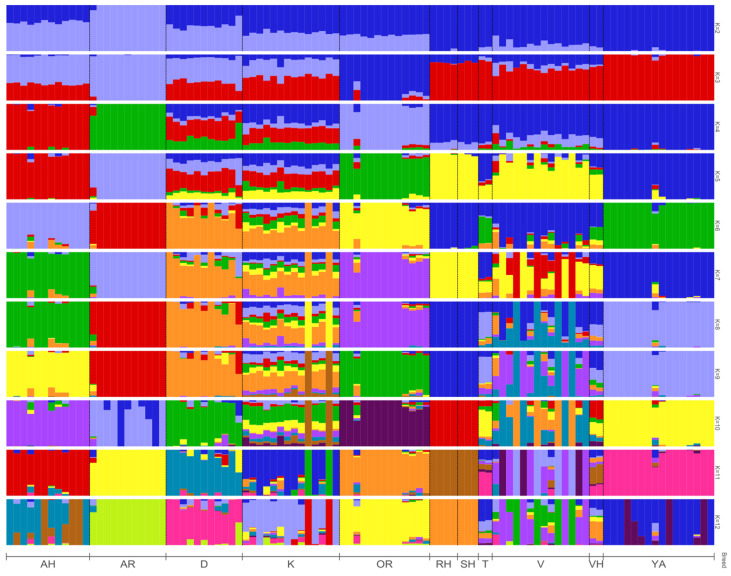
Comparative structure of equine populations calculated utilizing Admixture 1.3 software with the range of ancestral clusters K varying from 2 to 12. Breed designations: Arabian (AR), Akhal-Teke (AH), Don (D), Orlov Trotter (OR), Vladimir Heavy Draft (VH), Russian Heavy Draft (RH), Soviet Heavy Draft (SH), Kabardin (K), Yakut (YA), Tuva (T), Vyatka (V) breeds.

The analysis of over 50,000 SNP variants has uncovered the genomic structure of 11 horse breeds. ADMIXTURE analyses were conducted on the investigated horse groups, taking into account 2 to 12 ancestral clusters. The cross-validation error is shown in Figure 4. The smallest error value was not obvious, and was found to be K = 8. There was differentiation by horse groups based on utilisation. These are horses of riding and trotting breeds (AR, AH, D, K, OR), horses of heavy harness, and indigenous breeds (SH, RH, VH, V, T, YA), which clearly stood out at K = 2 (Figure 4 and Figure 5). At a value of K = 4, we could also distinguish separate groups of Arabian (AR), Akhal-Teke (AH), Orlov Trotter (OR), and Yakut (YA) horse breeds. At K = 8, most breeds were observed to be structurally homogeneous. However, the individuals of the Vyatka and Kabardin breeds were genetically heterogeneous. The Tuva equine breed exhibits resemblances to the Yakut breed. This similarity endures even as the quantity of potential ancestors increases. At K = 7, the similarity of the Don and Kabardin breeds was revealed. But already at K = 11 this similarity disappears.

**Figure 6:**
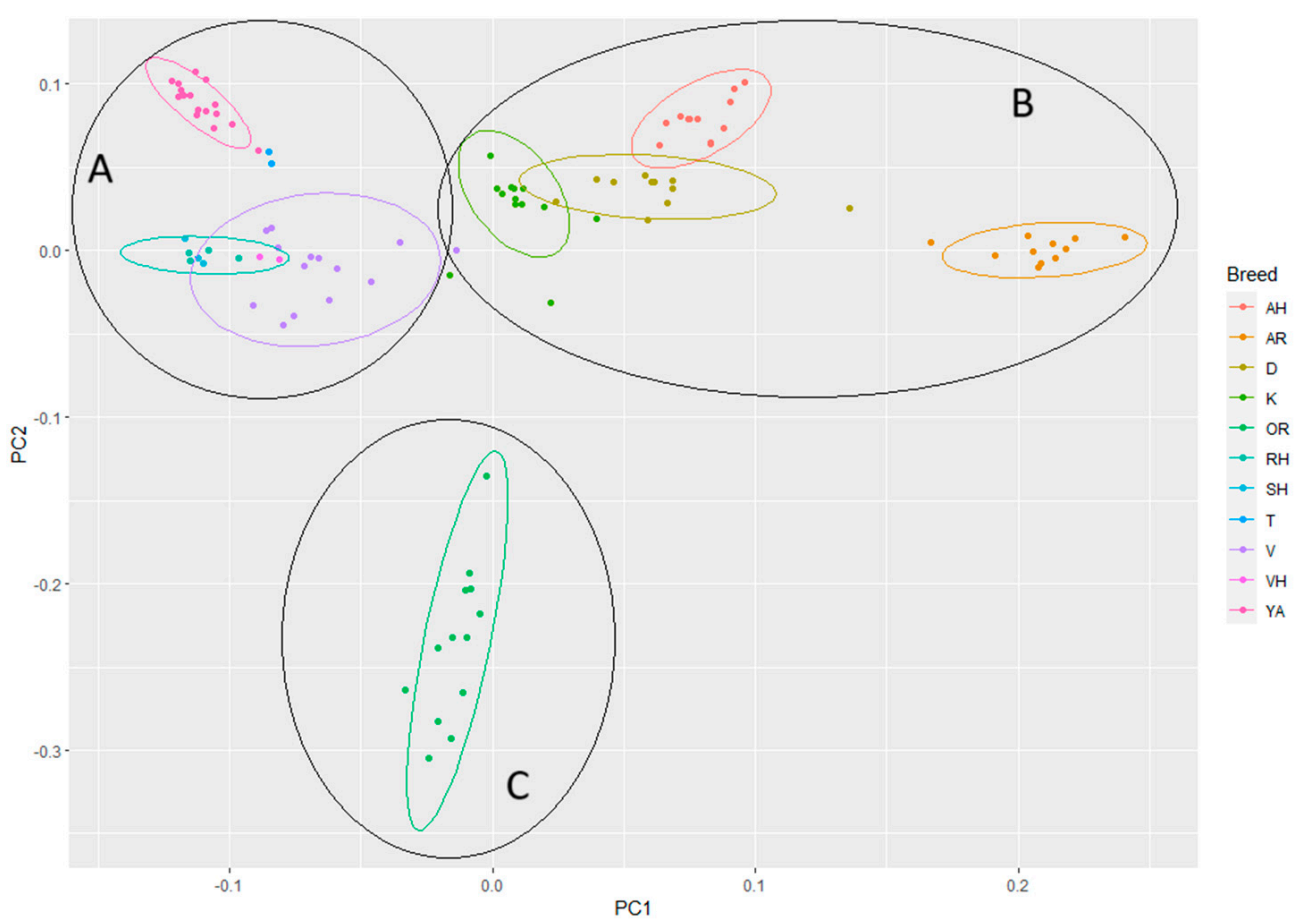
Genetic identification and clustering of populations through multivariate scaling in (A) riding horses; (B) draft horses, (C) Orlov Trotter. Breed designations: Arabian (AR), Akhal-Teke (AH), Don (D), Orlov Trotter (OR), Kabardin (K), Yakut (YA), Tuva (T), Vyatka (V), Vladimir Heavy Draft (VH), Russian Heavy Draft (RH), and Soviet Heavy Draft (SH).

PCA analysis has confirmed the segregation of horses on several blocks (Figure 6). A number of consolidated breeds have emerged, e.g.,: Yakut, Orlov Trotter, Arabian, and Akhal-Teke. This matches to data obtained from ADMIXTURE. Arrangement of the breeds according to vector 1 divided all studied breeds into two groups, as follows at ADMIXTURE analysis. In relation to the second vector, the Orlov Trotter breed stands out on one side, while the AH and YA breeds, bred in extreme conditions, stand out on the other side. The remaining breeds take up the central position.

Highly informative representation of horse number structure, displayed at K = 8.

## 4. Discussion

To date, horse breeding in Russia has four distinct directions of productivity including working purpose, food production, sports, and breeding. It is believed that breeds can be divided into coldblood (harness), warmblood (riding and trotting) breeds, and breeds for food production (milk, meat) [2,14,15]. Our studies have confirmed similar differences at the genome level.

Draft and indigenous horse breeds serve a dual purpose, as they can be used for both working purpose and production, including milk and meat production. They are bred in local areas, often with extreme climatic conditions [15,16,17]. In our study, they are represented by Vyatka, Tuva, and Yakut horse breeds, which are characterized by exceptional endurance, as well as high adaptability to the surrounding natural and climatic conditions. The body features of aboriginal horses include a medium-sized stature, broad body, elongated body, and relatively short legs. Scientific and technological advances have had a significant impact on the quantitative aspects of indigenous horse populations, and the decline in population size has led to a significant reduction in genetic diversity. However, all local breeds have a distinctive, and often unique, genetic structure, which in most cases is characterized by the presence of private alleles.

The Vyatka breed is an indigenous breed of northern forest type horses, established during the end of XVII—beginning of XVIII centuries in the modern Kirov region and Udmurtia territories [17]. It is believed that the Vyatka breed history begins with the Livonian kleppers arrival to the Vyatka region first in 1374 and later, be the decree of Peter I, around 1720. However, the strong and energetic horse was already being used in postal troikas—a traditional Russian harness driving combination consisting of three horses abreast—by the end of the XVIII century; thus, the participation of Livonian kleppers has not been proven yet. A considerable damage to the Vyatka gene pool occurred in the middle of the XX century, when draft horses, as well as Orlov and Russian trotters, were used for breed improvement. The genomic analysis, conducted in our study, confirms the historical establishment of this population. Moreover, the Vyatka breed genome clearly shows traces of similarities with Russian and Soviet Heavy Draft breeds. In addition, genomic analysis revealed accumulation of homozygosity in certain individuals, and Admixture analysis showed genetic heterogeneity of the population.

The Yakut horse is one of the oldest local breeds. It was formed in the conditions of the Far North of Yakutia several thousand years ago. It is the most cold-resistant indigenous breed, which has been developed via popular breeding under the strong influence of natural selection. In Yakutia, horses are kept outdoors all year round (at temperatures up to +40 °C in summer and −60 °C in winter), and have to search for food on their own. Traditionally, these horses are used for meat and milk production, as well as for riding. Currently, there are several types of Yakut horses in the Republic of Yakutia, which vary in their habitat, as well as in the extent to which they have been crossbred with other breeds [18]. Purebred animals from the central region of the Republic of Sakha (Yakutia) of the Russian Federation were used in our study. Genetic analysis revealed no traces of crossbreeding with other breeds (with the exception of one animal with low bloodlines from other breeds). We also identified low heterozygosity and high inbreeding levels in the studied population. The strong pressure of natural selection has significantly narrowed the genetic diversity, relatively to SNP pool, used in the analysis. Our results were also confirmed through the studies of other authors, in which 7 Yakut horses were studied using whole genome sequencing (WGS), and similar data were obtained, in particular, through FROH analysis.

The Tuva horse is another indigenous breed of the Russian Federation. This breed was also developed via popular breeding over many centuries in the Republic of Tuva territory. The unique climatic conditions of Tuva had a great influence on the development of the breed, which was formed without noticeable influence from other breeds until the mid-20th century. The Tuva horse is characterized as a highly efficient and enduring local breed, which easily withstands year-round grazing conditions. Moreover, this breed has a high working capacity, as well as good meat and milk productivity [7].

With the emergence of large cities, the growth of trade and cargo turnover prompted the need for a large, massive horse capable of carrying large loads, and pulling heavy agricultural implements and machines. In the first half of the 19th century, a fairly large group of draft horses was created in the Voronezh province. This group of horses was called Bityug. However, in the middle of the 19th century, heavy draft horses from European countries began to be imported to Russia in large numbers. These were Ardennes, Clydesdales, Shires, Suffolks, Percherons and Brabançons.

Through crossing Brabançons with local draft horses of various origins, the Soviet draft breed was developed. Horses of this breed have versatile working qualities, speed, strength, and endurance. They acclimatize well in harsh conditions, and are not demanding in terms of maintenance. This is the largest breed of heavy draft horses of Russian selection.

Russian draft horses are intended for agricultural work. In addition, they have high meat quality, as well as high milk productivity. This makes this breed very promising in productive horse breeding. The history of the creation of the breed is associated with the arrival of mountain-type Ardennes from Belgium to Russia that occurred in the mid-19th century. To increase the number of Ardennes, they were crossed with draft mares of different breeds. The main breeds used were Brabançons and Percherons. As a result of many years of breeding work through complex interbreeding of Ardennes and their crosses, the Russian draft horse breed was created in the mid-20th century. Horses of this breed are not large, with well-defined draft forms, wide-bodied, and have a strong constitution, as well as a balanced temperament. Currently, the Russian draft breed is the most common draft breed in Russia.

The Vladimir breed of horses was bred in the Vladimir and Ivanovo regions of Russia. The bulk of the horse population in these areas was made up of small peasant horses of strong constitution. They were hardy, but weak in strength. At the end of the 19th century, crossing of local mares with stallions of the Percheron, Suffolk, and Ardennes breeds began. This led to the gradual replacement of outbred horses with improved crossbreds. At the beginning of the 20th century, Clydesdale stallions had a significant influence on the formation of the breed, which contributed to the further enlargement of horses, and gave them a draft type. The creation of the breed was completed in the mid-20th century. The horses of the Vladimir draft breed are distinguished by their large stature, well-developed muscles, strong constitution, and energetic temperament. These are strong and hardy horses that are well adapted to the climatic conditions of Central Russia.

According to the data we received, heavy horse breeds are the closest to aboriginal breeds (Yakut, Tuvan, and Vyatka breeds). In our study, three breeds were used: the Soviet heavy truck, the Russian heavy truck, and the Vladimir heavy truck. Although no significant differences were found between the draft breeds, these breeds constitute a separate block, distinct from other breeds. Other studies of draft breeds are represented only through studies of individual breeds or the mitochondrial genome [19,20,21].

A number of horse breeds have a significantly lowered level of genetic diversity as a result of high selection pressure and purebred breeding. The Akhal-Teke horse, along with the Arabian and Thoroughbred, belongs to the purebred group of breeds. This horse breed was developed for riding, presumably about 5000 years ago, in the modern Turkmenistan territory (Ahkal-Teke), which explains its adaptation to dry and hot climates. Being the oldest of the purebred breeds, the Akhal-Teke horse has had a significant influence on the Arabian and Thoroughbred breeds. The limited use of stallions in breeding also significantly increased the population’s homozygosity level. The Akhal-Teke breed, along with the Arabian horse, has had a minimum effective population size for many generations, which is consistent with our results of significant homozygosity in both breeds. Moreover, our results are consistent with earlier studies that were conducted using Axiom™ Equine Genotyping Array on Russian horse breeds [8]. Other studies involving Arabian and Akhal-Teke breeds have also found high levels of homozygosity; however, given that they studied five individuals of each breed, it cannot be stated that the obtained data are sufficient for a general breed characterization [22,23].

Other authors have studied the origin of Akhal-Teke horses [24]. The data obtained on ADMIXTURE and PCA of Syrian Arabian horses and Akhal-Teke horses are fully consistent with our data, and confirm the origin of the Akhal-Teke breed from eastern horses.

Riding horses are performance-oriented; therefore, these breeds are occasionally improved by thoroughbreds. ADMIXTURE and PCA results revealed differences between individuals in riding horses (particularly Kabardin and Don breeds), which may be the result of introgression from other breeds. Other studies emphasize that the loss of local breeds’ genetic uniqueness is due to the improvement of their sports performance by purebreds [8,24,25].

The Kabardin breed is one of the oldest breeds, used both for riding and harness. A unique characteristic they possess is their adaptability to mountainous landscapes [26]. It is believed that the breed is the result of complex and lengthy crossing of the horses of steppe breeds with eastern ones, and breeding in the specific mountain conditions of the North Caucasus. In the 16th century, horses of the Kabardian breed were already famous far beyond the Caucasus. Kabardian horses are very hardy, unusually brave, and careful when riding along mountain trails. Horses of this breed have a more advanced vestibular apparatus, and strong hoof horn, which is especially important for long rides in mountainous areas. They are characterized by a strong constitution, and a good ability to quickly restore body condition and maintain it in the winter when kept in herds. Our study identified unique genotypes that need to be conserved.

The Don breed is also an old breed, used both for riding and harness, which was developed during the XVIII-XIX centuries in the territory of the modern Rostov region. The first stud farm of the Don breed was established by the Cossack ataman M.I. Platov back in 1770, on the left bank of the Don River. The ancestor of this eastern steppe horse is the Kalmyk or Mongolian horse; for its improvement, stallions of Persian, Kabardin, Turkmen, Karabakh, Nogai, and Turkish selection were used, and from the beginning of the XIX century, the Arabian and Thoroughbred breeds as well. The Don breed was strictly selected for its cavalry qualities, endurance, and adaptability [27]. Nature has endowed the Don breed with a very strong constitution, unpretentiousness, good distance abilities, undemanding conditions for keeping and feeding, and high performance. Modern horses of the Don breed are characterized by good conformation, dry strong limbs, and strong hooves. There are several intra-breed types in the Don breed, which expands the boundaries of the use of Don horses. Don horses, especially, had an impact on horse breeding in Kazakhstan and Kyrgyzstan. There is a significant proportion of Don blood in the Kushum, Kustanai, and Novokyrgyz breeds. As an improver of local horses, Don horses were widely used in the Lower Volga region, the Urals, the mountainous part of Altai, Tyva, Buryatia, the Chita region, and the North Caucasus. Both ADMIXTURE and PCA analysis revealed the similarity of Don horse with the Kabardin breed, and for some animals with the Arabian breed. Earlier studies revealed traces of Thoroughbreds in Don stallions [8].

The Orlov Trotter formed a separate cluster in both structural and PCA analyses. According to the second ADMIXTURE vector, this breed differs significantly from the studied breeds. Orlov Trotter is a famous Russian light harness horse breed with the hereditary ability to trot. The breed was developed in Russia, in the second half of the XVIII—early XIX centuries in the Khrenovskoy stud farm in Voronezh province under the leadership of its owner Count A.G. Orlov through a complex crossbreeding method using Arabian, Danish, Dutch, Mecklenburg, and other breeds. However, at the end of the XIX century, mass mestization began, during which some of the Orlov mares were mated with American trotting stallions. In the 20th century, large losses in Russian horse breeding were associated with the Civil War. At this time, most of the breeding stock was lost. In this connection, the Orlov Trotter was on the verge of extinction. However, already in the middle of the 20th century, the Orlov Trotter became one of the most numerous breeds in Russia. In order to increase the trotter speed, and expand the genetic diversity in the breed, crossing of Orlov trotting mares with Thoroughbred horses, standardbred and Russian trotting stallions, was carried out in the second half of the 20th century (in the 1960s). As a result of this crossing, the level of heterozygosity of the population increased, without causing significant changes in the number of alleles found in the Oryol trotting breed [27]. Our early research [8] on ADMIXTURE found traces of Thoroughbred and Standardbred breeds. To date, purebred breeding of the Orlov Trotter is under strict control, and the infusion of blood from other breeds is forbidden. The Orlov Trotter is the pride of Russia, its national treasure and calling card. The Orlov Trotter breed in its modern form is the result of long-term breeding work of many generations of the breeders. However, due to the small population size of the breed, we found high FROH in all animals analyzed. However, in PCA we see high inter-animal variability in the second component. This may be due to small introductory crosses at some historical stages of breeding.

## 5. Conclusions

Scientific and technological progress, along with historical factors, have had a significant impact on indigenous horse populations. The reduction of population size has led to a significant decrease in genetic diversity, and the use of purebreds for improvement has led to a decrease in divergent breed traits. To date, the problem of maintaining the genetic diversity in horses is of great importance. There is a risk of permanently losing not only the horse breeds themselves, but also the monuments of the historical development of mankind, that include not only artworks and architecture, but also the unique genomic architecture of animal breeds. A balanced approach is required when using the current horse gene pool, as it affects the preservation of national traditions and history. Our research helps to further evaluate the problems of genetic variability and inbreeding in horse breeds. The search for traces of selection helps to expand the understanding of genetic factors contributing to the adaptability of horses to extreme environments.

## Figures and Tables

**Table 1:** Values (M ± SE) of the observed heterozygosity (Ho), expected heterozygosity (He) and inbreeding coefficient (FIS) calculated from all SNPs for the seven horse populations. Breed designations: Arabian (AR), Akhal-Teke (AH), Don (D), Orlov Trotter (OR), Kabardin (K), Yakut (YA), Vyatka (V) breeds.

Breed	Number of Samples	Ho	He	FIS
AH	12	0.329 ± 0.001	0.310 ± 0.001	−0.05 ± 0.001
AR	11	0.303 ± 0.001	0.294 ± 0.001	−0.027 ± 0.001
D	11	0.342 ± 0.001	0.325 ± 0.001	−0.048 ± 0.001
K	14	0.342 ± 0.001	0.328 ± 0.001	−0.039 ± 0.001
OR	13	0.309 ± 0.001	0.294 ± 0.001	−0.043 ± 0.001
V	14	0.321 ± 0.001	0.302 ± 0.001	−0.054 ± 0.001
YA	16	0.298 ± 0.001	0.288 ± 0.001	−0.030 ± 0.001

## Data Availability

Data are contained within the article.
